# Effect of Berberine Isolated from Barberry Species by Centrifugal Partition Chromatography on Memory and the Expression of Parvalbumin in the Mouse Hippocampus Proper

**DOI:** 10.3390/ijms22094487

**Published:** 2021-04-26

**Authors:** Radosław Szalak, Wirginia Kukula-Koch, Małgorzata Matysek, Marta Kruk-Słomka, Wojciech Koch, Lidia Czernicka, Daariimaa Khurelbat, Grażyna Biała, Marcin B. Arciszewski

**Affiliations:** 1 Department of Animal Anatomy and Histology, Faculty of Veterinary Medicine, University of Life Sciences, 12 Akademicka Str., 20-950 Lublin, Poland; malgorzata.matysek@up.lublin.pl (M.M.); mb.arciszewski@wp.pl (M.B.A.); 2 Chair and Department of Pharmacognosy, Medical University in Lublin, 1 Chodźki Str., 20-093 Lublin, Poland; 3 Department of Pharmacology and Pharmacodynamics, Medical University of Lublin, 4a Chodźki Str., 20-093 Lublin, Poland; marta.kruk@umlub.pl (M.K.-S.); grazyna.biala@umlub.pl (G.B.); 4 Chair and Department of Food and Nutrition, Medical University of Lublin, 4a Chodźki Str., 20-093 Lublin, Poland; kochw@interia.pl (W.K.); lidia.czernicka@umlub.pl (L.C.); 5 Department of Pharmaceutical Chemistry and Pharmacognosy, School of Pharmacy, Mongolian National University of Medical Sciences, Zorig Str., Ulaanbaatar 14210, Mongolia; daariimaa@mnums.edu.mn

**Keywords:** *Berberis sibirica* Pall., Berberidaceae, berberine, calcium-binding proteins, parvalbumin, hippocampus, memory and learning, mice, counter-current chromatography

## Abstract

Neurodegenerative diseases associated with memory disturbances are important health issues occurring due to a prolonged life span. This article presents the results of a study targeting the emergence of a drug candidate with antiamnesic properties. The effect of berberine (BBR), an isoquinoline alkaloid isolated from the overground parts of *Berberis sibirica* Pall., on memory and expression of parvalbumin in the mouse hippocampus proper were determined. High-purity BBR was isolated by centrifugal partition chromatography from a methanolic extract from *B. sibirica* by using a methyl-*tert*-butyl ether and water (1:1 *v*/*v*) solvent system with 10 mmol/L of triethylamine and hydrochloric acid. In an in vivo study, we assessed the influence of the chronic administration of BBR on different stages of memory-related responses in mice. Our results indicated that the chronic administration of BBR in a higher dose (5 mg/kg) improves long-term memory acquisition in mice, as determined in the passive avoidance test. The hippocampal CA1–CA3 fields showed an increased number of parvalbumin-immunoreactive neurons (PV-IR) and nerve fibers as compared to the control. No significant changes in the dentate gyrus were observed between the groups. The HPLC-ESI-QTOF-MS/MS analysis of the biological material revealed the content of BBR as 363.4 ± 15.0 ng (4.11% of RSD) per brain, 15.06 ± 0.89 ng (5.91% of RSD) per hippocampus, and 54.45 ± 1.40 (4.05% of RSD) ng in 100 µL plasma. The study showed that BBR could be a factor influencing the expression of PV in hippocampal neurons. We speculate that BBR may modulate the level of Ca^2+^ in neurons and thus potentially act as a neuroprotective factor against neuronal damages.

## 1. Introduction

Neurodegenerative diseases are characterized by the deterioration of cognition that frequently occurs in senile patients. There is a direct relationship between an increasing life expectancy supported by a better access to medicine and the number of patients suffering from dementia. According to the World Health Organization, the number of people suffering from Alzheimer’s disease (AD) is increasing rapidly. It is estimated that the number of patients with AD will increase from approximately 36 million to 115 million by 2050 [1]. The features of memory impairment occurring during AD progression include not only memory loss but also behavioral changes in patients, cognitive impairment, or even death. These alterations are induced by a disturbed homeostasis of dopaminergic, monoaminergic, and serotoninergic neurotransmission that affects some areas of the brain.

Hippocampus proper (HP) is a brain structure that forms part of the limbic system. HP, which consists of Ammon’s horn (CA) and the dentate gyrus (DG), is a peculiar brain memory and reasoning center in which the sensory, emotional, and cognitive elements are interrelated [2]. HP is, on the one hand, a very plastic area of the brain and, on the other hand, a very sensitive structure prone to injury or trauma [3,4].

In the course of neurodegenerative diseases, accelerated aging of the brain and irreversible changes in the structures of the central nervous system (CNS) occur [5]. In the hippocampus, neuronal deficiencies, atrophy of nerve fibers, contraction of the entire structure, and ultimately its atrophy are observed [6,7]. The consequence of these changes is cognitive impairment [8]. In neurodegenerative processes, there is a toxic increase in the intracellular levels of Ca^2+^ that modifies neuronal functions such as synaptic plasticity, metabolism, or the transfer of information between different organelles. Calcium-binding proteins (CaBPs), and among them parvalbumin (PV), are the first line of defense because of their buffering properties, which allow nerve cells to rapidly achieve homeostasis [9,10]. PV exhibits a high Ca^2+^-binding capacity and is referred to as a slow calcium buffer [11,12]. In the CNS, PV is present in the GABA-ergic neurons of the hippocampus [13], and the PV interneuron dysfunction is reported to be associated with AD [14,15]; however, it remains unclear how the PV interneuron function is altered in early AD or how these changes contribute to AD progression. The treatment of neurodegenerative diseases is difficult. Acetylcholinesterase inhibitors, the first choice of drugs for treating dementia, can only slow down the progression of the disease by increasing the concentration of acetylcholine (Ach) in the synaptic cleft and, by this action, stimulating the memory processes. The currently available therapeutic strategies are, however, burdened with numerous limitations: the emergence of tolerance to the doses used, insufficient effects, or the occurrence of unpleasant side effects. In view of the above-mentioned findings, there is a need to continue the search for new drug candidates that will not only inhibit the decomposition of Ach in the synaptic cleft, but also increase the number of neurons and stimulate their physiology. The presented work aims to determine the impact of berberine—a well-studied acetylcholinesterase inhibitor, widely distributed in plants—on the intracellular calcium ion levels and the number of PV- immunoreactive (PV-IR) neurons. The additional pharmacological action could support neuronal functions in patients with progressive neurodegeneration. This task is important considering the fact that the number of people suffering from memory disorders is increasing every year. Berberine (BBR) is a representative compound of isoquinoline alkaloids present in several plants—from Berberidaceae, Papaveraceae, Menispermaceae, Ranunculaceae, and other botanical families—that shows antibacterial, bile-production enhancing, and anti-inflammatory activities [16,17]. Recent findings have also confirmed the beneficial properties of BBR for the CNS diseases, including dementia, AD, and epilepsy, which proves the ability of BBR to cross the brain-blood barrier when administered to animals or patients [18]. Although BBR has been proved to exhibit cognition-enhancing properties in previous studies [19], its mechanisms of action still require a deeper understanding.

The present study aimed to determine whether BBR, an isoquinoline alkaloid present in several plant species including the Siberian barberry (*Berberis sibirica* Pall.), administered intraperitoneally to mice at the dose of 2.5 and 5 mg/kg, affects the expression of PV-immunoreactive neurons (PV-IR) in the CA1–CA3 fields of the hippocampus and in the DG, and whether it participates in the improvement of memory processes. To understand the background of its therapeutic effects in a more comprehensive manner, the effect of the administered alkaloid on memory will also be investigated in behavioral studies on mice, and the level of BBR in their brain and plasma will be estimated by chromatographic studies using a high-performance HPLC-ESI-QTOF-MS platform.

## 2. Results

### 2.1. Isolation of BBR from Barberry Extract

**Figure 1:**
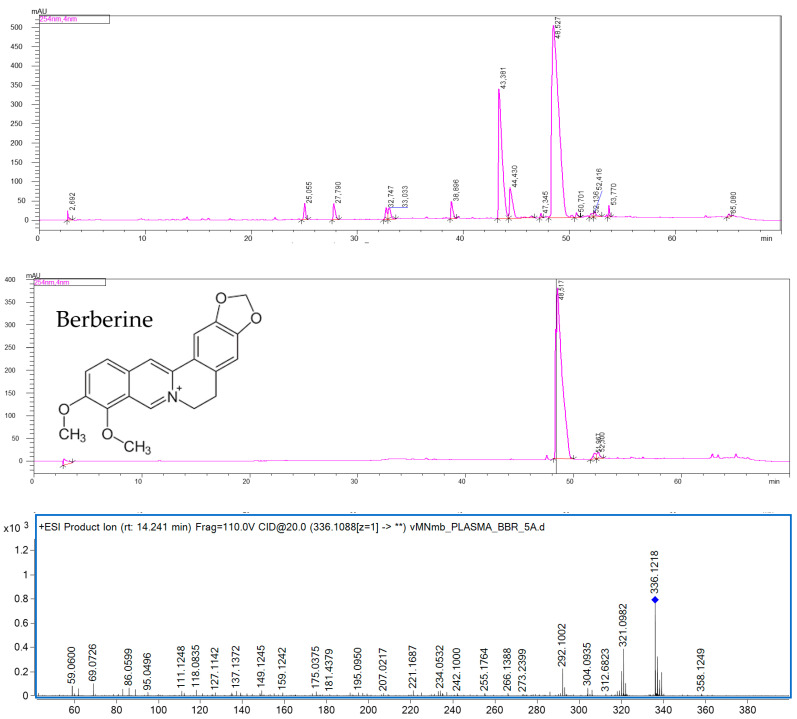
HPLC-UV chromatogram of the total extract from *Berberis sibirica* (**top**), the purity of the isolated berberine recorded at 254 nm (**middle**), and the fragmentation pattern (MS/MS chromatogram) of berberine (**bottom**).

Centrifugal partition chromatography (CPC) was used to isolate BBR from the methanolic extract obtained from the overground parts of *B. sibirica* and led to the recovery of the alkaloid with high purity, exceeding 96%—directly from the total extract (see Figure 1) [20].

A high purity of the alkaloid was achieved because of the application of a pH-zone refining mode of operation that is based on the addition of a base and an acid to the biphasic solvent system [20]. The presence of an eluter and a retainer helped to obtain sharper peaks of alkaloids than that obtained in the elution-extrusion mode of separation, which is the most commonly used one in the fractionation of natural products. The pH-zone refining mode uses the ability of alkaloids to be present in two forms: unpolar bases and polar salts. During the separation process, the nitrogen-containing compounds can change their forms depending on the pH of the phase, and because of these properties, they are separated from other constituents characterized by a different chemical property. The elaborated isolation protocol provided a sufficient quantity of BBR for in vivo studies on mice.

The recorded UV, MS, and fragmentation spectra of the isolated compound resembled those reported in the scientific literature and presented in the former studies of the authors, which led to the confirmed identification of BBR [17]. Under the applied conditions, BBR was easily ionized by an electrospray ionization mechanism. The applied voltage settings and other parameters provided two clear MS/MS fragments at the *m*/*z* values of: 321 and 292—from the subsequent de-attachments of methoxyl groups from the molecular ion, and 304—from the loss of the methylene bridge, which is a characteristic of BBR structure.

### 2.2. Influence of Chronic Administration of BBR on Memory Acquisition in Mice in the Passive Avoidance Test

**Figure 2:**
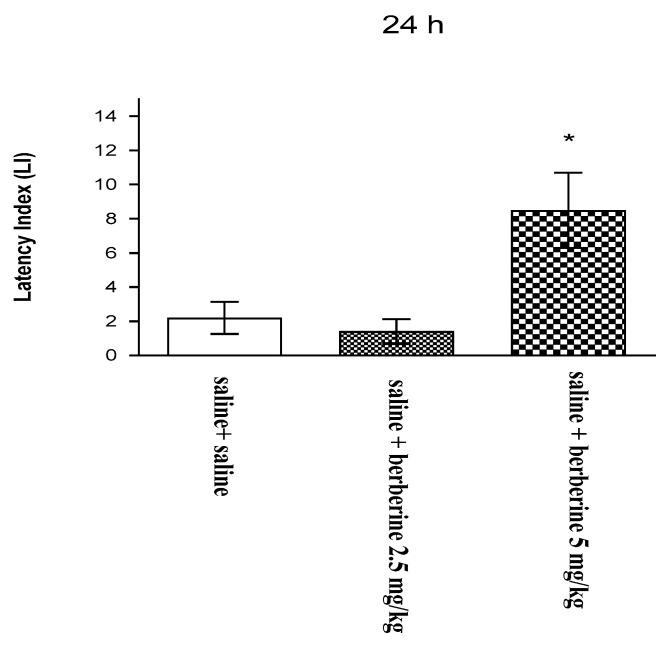
Effects of chronic berberine administration on the latency index (LI) during the memory acquisition trial with the PA test in mice. Berberine (2.5 or 5 mg/kg; i.p.) or saline was administered for 7 days. On the 8th day, 15 min after the last injection, the first trial was conducted. After 24 h, the mice were re-tested; *n* = 7–9; mean ± SEM; * *p* < 0.05 vs. saline-treated control group; Tukey’s test.

One-way ANOVA showed that the chronic intraperitoneal (i.p.) administration of BBR (2.5 and 5 mg/kg) had a statistically significant effect on latency index (LI) values for memory acquisition [F(2,22) = 7038; *p* = 0.0049]. Indeed, chronic treatment with BBR at the dose of 5 mg/kg significantly increased LI values in mice in the passive avoidance (PA) test compared to that in the saline-treated control group (*p* < 0.05, post-hoc Tukey’s test (Figure 2)), indicating that a chronic administration of BBR at this dose improves the acquisition of memory and learning in mice in the PA test.

### 2.3. Immunohistochemistry

**Figure 3:**
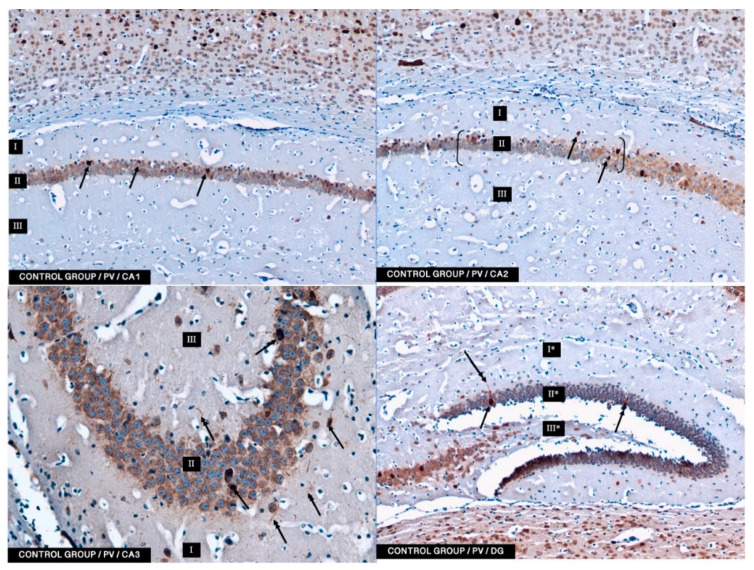
Control group. The immunoreactivity of the PV-IR of moderate neurons of CA1–CA3 mouse hippocampal fields and DG of the hippocampus is observed. (**I**: the marginal layer, **II**: the pyramidal layer, **III**: the multiform layer; the arrows indicate PV-IR neurons (**single arrow**) and PV-IR fibers (**double arrow**) of the hippocampus; magnification ×20).

**Figure 4:**
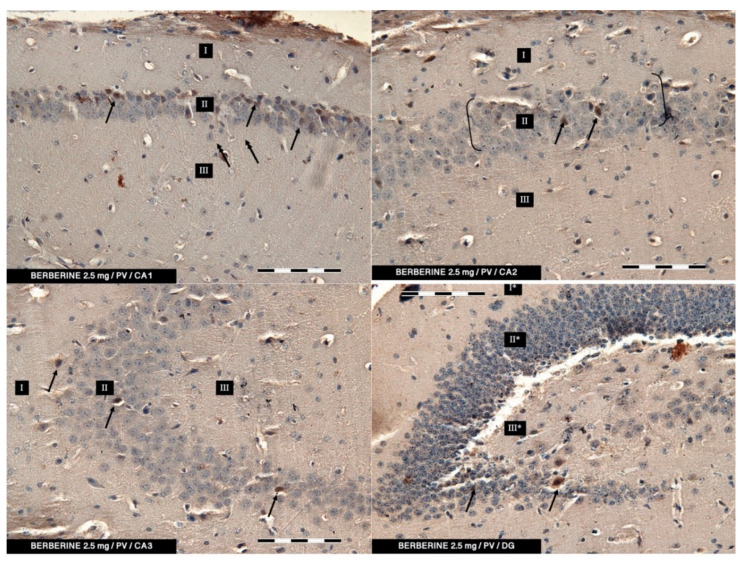
The experimental group treated with 2.5 mg/kg (i.p.) of BBR. The immunoreactivity of the PV-IR of moderate neurons of CA1–CA3 mouse hippocampal fields and DG of the hippocampus is observed. (**I**: the marginal layer, **II**: the pyramidal layer, **III**: the multiform layer; the arrows indicate PV-IR neurons (**single arrow**) and PV-IR fibers (**double arrow**) of the hippocampus; magnification ×20).

**Figure 5:**
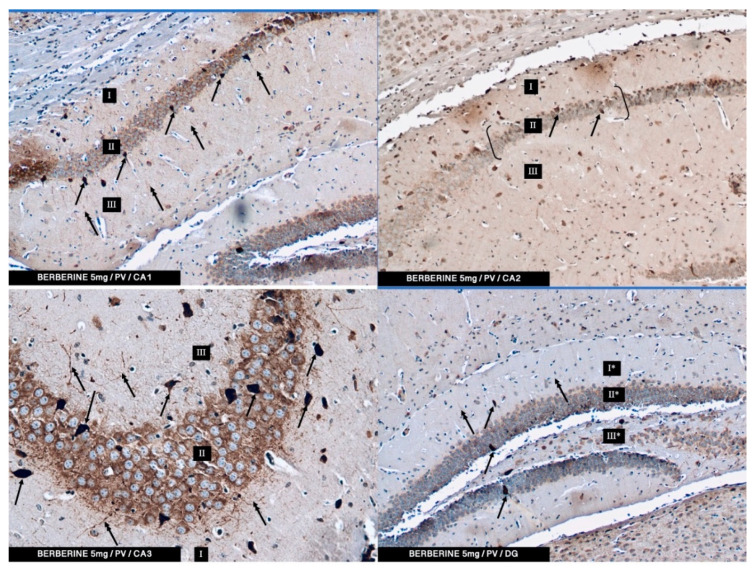
The experimental group treated with 5 mg/kg (i.p.) of BBR. The immunoreactivity of the PV-IR of moderate neurons of CA1–CA3 mouse hippocampal fields and DG of the hippocampus is observed. (**I**: the marginal layer, **II**: the pyramidal layer, **III**: the multiform layer; the arrows indicate PV-IR neurons (**single arrow**) and PV-IR fibers (**double arrow**) of the hippocampus; magnification ×20).

According to the results of the herein described studies, in both the control group and the experimental groups (2.5 and 5 mg/kg i.p. administration), immunoreactivity to PV (PV-IR) was found in a relatively high number of neurons in all the studied fields of the mouse hippocampus (CA1, CA2, and CA3) as well as in neurons of DG. PV-IR neurons were observed in the marginal, pyramidal, and multiform layers in all CA fields and in molecular and granular layers and hillus of DG (Figure 3, Figure 4 and Figure 5). Neurons were characterized by the presence of cytoplasmic and nuclear reactions. Their cytoplasm, however, was repeatedly more intensely stained than their nuclei. Oval, round, and triangular neurons with a moderate (++) and intense (+++) PV-IR reaction were observed in the CA1–CA3 fields and DG in the control group (Figure 2) and in the 5 mg/kg experimental group (Figure 5). In contrast, in the 2.5 mg/kg experimental group, a only moderate (++) to weak (+) reaction in PV-IR neurons was visible (Figure 4). Additionally, after the i.p. administration of 5 mg/kg BBR, the immunoreactivity to PV was observed in nerve fibers emerging from PV-IR neurons (especially in the CA1 and CA3 hippocampal fields), while in DG the immunoreactivity was observed only in a few PV-IR neurons and single dendritic fibers (Figure 5). In the control group, single PV-IR nerve fibers were observed in the CA3 and DG, whereas in the 2.5 mg experimental group, few PV-positive dendritic fibers were found only in CA1 (Figure 3 and Figure 4). A significant increase in the number of PV-IR neurons in all the hippocampal fields was observed after the administration of 5 mg/kg BBR as compared to both the control group and the 2.5 mg/kg experimental group. No significant differences were observed in the number of PV-IR neurons in the CA1 and CA3 fields of the hippocampus between the control group and the 2.5 mg/kg experimental group, whereas the 2.5 mg/kg experimental group showed a slight decrease in the number of PV-IR neurons in the hippocampal CA2 field as compared to the control group and the 5 mg/kg experimental group (Figure 3, Figure 4 and Figure 5).

In DG, no significant differences were observed in the populations of PV-IR neurons between the 2.5 and 5 mg/kg experimental groups and the control group. The average numbers of PV-IR neurons found in the fields of the hippocampus and in the DG of the control and the 2.5 and 5 mg/kg experimental groups are summarized in Table 1.

### 2.4. Results of the Quantitative Study

**Figure 6:**
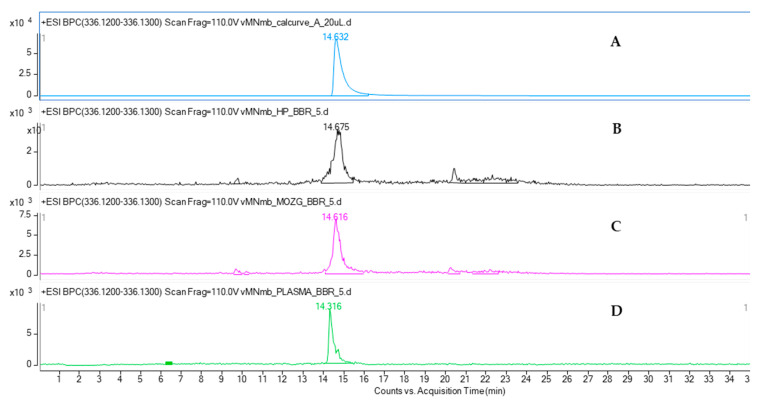
Total ion chromatogram of berberine standard (**A**) and the extracted ion chromatograms (EIC) of berberine peak in the hippocampus (**B**), brain (**C**), and plasma (**D**), recorded in the positive ionization mode.

The amount of BBR in the biological samples was quantified by drawing a calibration curve for the BBR standard tested in a linear range of concentrations. The applied chromatographic conditions provided clear data on the presence of BBR in the tested biological samples (Figure 6): in the hippocampus, brain, and plasma. All samples were analyzed in the same time span from the administration of 2.5 and 5 mg/kg b.w. of BBR, i.e., after 4 h. The concentration of BBR in the brain was found to exceed its content in the plasma after this time. The analysis revealed that the concentration of BBR in the brain was 363.4 ± 15.0 ng (4.11% of RSD) per brain for 5 mg/kg b.w. of BBR administration and 0.1528 ± 0.0071 ng (4.65% of RSD) for 2.5 mg/kg b.w. of BBR administration. The content of this alkaloid in the hippocampus estimated for the 2.5 and 5 mg/kg b.w. administration was 15.06 ± 0.89 ng (5.91% of RSD) and 38.12 ± 2.12 ng (5.55% of RSD) per extracted organ, respectively, and 26.01 ± 0.6 (2.31% of RSD) ng and 54.45 ± 1.40 (4.05% of RSD) ng in 100 µL plasma, respectively.

## 3. Discussion

In our present study, BBR was isolated from the overground parts of *B. sibirica* collected in Mongolia by centrifugal partition chromatography.

Structural similarities between the molecules belonging to isoquinoline alkaloids and their wide distribution in the extracts of numerous plant species pose a challenge for the development of favorable conditions for chromatographic separation using different separation techniques. Centrifugal partition chromatography, that belongs to the hydrostatic type of counter-current chromatography, is a modern isolation technique whose principle is based on the fractionation of complex samples between two immiscible solvents [21]. By using this technique in the fractionation of isoquinoline alkaloid-containing plant extracts, their tailing process in the column and their adsorption on the stationary solid phase, which occurs when using traditional chromatographic techniques with solid adsorbents, were successfully limited, resulting in a marked increase in the isolation efficiency.

Isolation of isoquinoline alkaloids by counter-current chromatographic separation has been developed by different researchers. In their studies, several different biphasic solvent systems were used to achieve a highly efficient recovery [20,22,23,24]. In the present study, a mixture of polar solvents: methyl-*tert*-butyl ether and water (1:1 *v*/*v*) with an addition of 10 mmol/L HCl and triethylamine was found to effectively fractionate the analyzed extract from *B. sibirica* overground parts [20].

Among the isoquinoline alkaloids, BBR is certainly one of the best studied representatives. Previous studies have confirmed the ability of BBR to cross the blood-brain barrier and induce effects on the CNS. An interesting aspect is that the pharmacological effect of this alkaloid on the nervous system was first reported in the 1970s [25]. Since then, the therapeutic effect of BBR has been widely studied in various neurological diseases, including memory disturbances and AD-related symptoms.

In our studies, this natural compound was proved to have a supporting effect on the memory process. Our previous findings revealed that an acute administration of BBR (5 mg/kg i.p.) significantly improved long-term memory acquisition [19]. On the basis of this effect, we assessed the influence of BBR on these memory stages after chronic administration. In the present experiment, we investigated for the first time the influence of the chronic administration of BBR on the memory-related responses in mice. To assess the cognitive function after BBR administration, we used the PA test, which is commonly used in pharmacological studies. We observed that the chronic administration of BBR at a higher dose (5 mg/kg i.p.) improves long-term memory acquisition in mice in the PA test.

These results agree with those of other available studies describing the positive influence of BBR on memory impairment in various animal models. BBR exerted strong effects to prevent memory impairment in mechanistically different amnesic animal models, including the transgenic mouse model of AD and the scopolamine- or streptozotocin-induced memory impairment model. The memory performance of rodents was observed in various behavioral tests that assess memory targeting effects and learning processes in rodents, e.g., water maze test, Y-maze test, or elevated plus maze test [26,27,28]. The observed effects suggested an overall improvement of memory function by BBR.

The BBR-induced memory improvement may be due to the complex mechanism of action of this compound, which includes neuroprotection through the cholinergic mechanism, anti-inflammatory effects, reduction of oxidative stress, and other mechanisms. One of the procognitive mechanisms of BBR observed in animal models of AD is related to the inhibition of the hyperphosphorylation of the Tau protein and production of Aβ—the two crucial proteins involved in the pathophysiology of AD [29]. According to previous studies, another possible mechanism of the anti-AD effects of BBR may be related to its inhibitory effects on key enzymes involved in the pathogenesis of AD: cholinesterase (ChE) or monoamine oxidase [30]. In the context of our results showing the procognitive effects of the chronic administration of BBR, the influence of this alkaloid on the cholinergic transmission seems to be the most important one. The cholinergic system plays a key role in the formation of memory pathways through cholinergic receptors and the neurotransmitter ACh. The decrease in cholinergic activity is responsible for memory disorders, occurring in many diseases, e.g., in AD. This decrease in cholinergic activity is associated with AD symptoms, and the improvement of cholinergic activity relieves AD symptoms, including memory-related disturbances. ChE is the major enzyme responsible for ACh destruction, and its inhibition results in the increased ACh levels in the brain. Therefore, many anti-AD pharmacological studies have focused on ChE inhibitors to attenuate cognitive disorders. In the scientific literature, several studies have shown the influence of BBR on cholinergic transmission. For example, chronic treatment with BBR (25–100 mg/kg) improved memory and learning performance and lowered oxidative stress and ChE activity in ethanol-treated rats tested with the Morris water maze paradigm [31]. These results, that agree with our experimental results, suggest that BBR has a positive influence on memory and learning processes and might be a potential therapeutic agent that prevents or delays the progression of AD or other memory-related disturbances.

The above-demonstrated results on the influence of BBR on memory of mice in behavioral tests can be explained by quantitative and molecular studies and by the influence of BBR on the expression of PV.

The quantitative analysis of mice plasma, brain, and hippocampus by HPLC-ESI-QTOF-MS chromatography confirmed the presence of BBR in all the tested samples. BBR has been shown to cross the blood-brain barrier. Although the majority of pharmacokinetic studies have been performed after an oral administration of BBR-containing preparations, the calculated data confirmed the presence of this alkaloid in brain cells [32]. The results of the present study are consistent with the previously reported quantitative data on the plasma and hippocampus levels of the studied compound. According to Wang et al. [33], the content of BBR in rats’ plasma was found to be at a similar level as that in the HP after intravenous administration. The authors reported that the BBR-containing preparation with 3 mg/kg of BBR had the highest plasma content, of 38.5 ng/mL, whereas the HP concentration was 30.7 ng/mL. Our results show a similar tendency. BBR was proved to be well distributed in animal tissues, including the brain. Qi et al. [34] compared the bioavailability of nine major constituents of a Huanglian Jiedu decoction that was also composed of a BBR-containing *Coptis chinensis* rhizome. In their HPLC-MS/MS-based quantitative studies, the concentration of BBR was the highest in the hippocampus, cortex, and striatum of rats.

Additionally, on the basis of the performed calculations, we can assume that BBR is present in the hippocampus at a much greater concentration than that in the remaining parts of the brain. The weight of mouse brain is estimated to be 3% of its total body weight, whereas that of the mouse hippocampus is only 0.06% of its total body weight. From these results, it can be observed that the difference in the concentration of BBR in the brain and hippocampus is only 10-fold: 363.4 ± 15.0 ng in the brain to 38.12 ± 2.12 ng in the HP.

In this preliminary study, the expression of PV in the hippocampal fields of mice was assessed to investigate the effects of BBR on immunoreactive neurons of the memory-related CNS structure. Recent findings show that BBR may protect nerve cells against oxidative stress and thus delay the aging process [35]. As mentioned above, the effectiveness of BBR in neurodegenerative diseases such as AD has been proved by different authors [35,36]. However, there is still less information on the influence of BBR on the CNS structures, especially those related to memory and cognition. Ca^2+^ ions are necessary for the proper functioning of neurons in the CNS. Calcium, in addition to its primary role as a mediator of cell signaling, is also involved in long-term processes such as memory acquisition [37]. This is possible through the interaction of Ca^2+^ with intracellular CaBPs. Some of these proteins, such as PV, were proved to be useful neuronal markers for various functional brain systems and their circuits. PV is known to buffer intracellular Ca^2+^, which contributes to the maintenance of synaptic properties.

It is presumed that altered CaBP levels or protein modification due to various reasons may lead to the impairment of calcium homeostasis in cells and, subsequently, to pathological conditions. It is assumed that neurons containing intracellular calcium bonds may have a greater Ca^2+^ buffering capacity and therefore should be more resistant to degeneration. Each modification of these proteins changes the normal level of intracellular calcium and causes the neuron’s death. These changes may result from aging, diabetes, or neurodegenerative diseases. The available literature provides information on the varying expression of PV under pathological conditions. Lee et al. [38] reported the loss of PV-IR neurons in the aging hippocampus of Mongolian gerbils. The decrease in the number of PV-IR neurons is more pronounced in the CA1 field than in the CA2 and CA3 fields, and also in DG. In contrast, Brady and Mufson [39] observed an approximately 60% decrease in the number of PV interneurons in the DG and CA1 and CA2 hippocampal fields of people with AD. Interestingly, according to their study, the number of PV neurons did not decrease in the CA3 field of the hippocampus as compared to that in the control group. On the other hand, in epilepsy, a loss of human hippocampal PV-IR cells, mainly in the CA1 and CA3 fields, was observed, while the PV-IR neurons of the CA2 field were less susceptible to damage [40].

In the present study, PV-IR neurons were observed in all hippocampal fields (CA1–CA3) and in the DG of mice. The average number of PV-positive neurons was increased in the experimental group with 5 mg/kg i.p. BBR administration as compared to the control group and the experimental group with 2.5 mg/kg i.p. BBR administration. A PV-IR reaction was also found in nerve fibers, and the level was markedly higher in the experimental group with 5 mg/kg i.p. BBR administration, while only single dendritic fibers were detected in the control group and the experimental group with 2.5 mg/kg i.p. BBR administration.

In the DG of all the tested groups, no increase was observed in the number of PV-IR neurons. Few single PV-IR nerve fibers were visible in the control and 5 mg/kg experimental groups, whereas no PV-positive dendritic fibers were found in DG of the 2.5 mg/kg experimental group.

PV occurs in interneurons (inhibitory neurons), which make up about 20% of all cortical neurons. The neurons expressing PV-IR are termed “fast-speaking interneurons”. As explained by Trachtenberg [41], “PV cells are not altering the computations performed by local excitatory neurons but altering their firing rates”. Thus, PV cells are ideally suited to control the cortical response. In this study, an increase of PV-IR neurons after the administration of BBR may suggest the effect of this alkaloid on neuronal Ca^2+^ metabolism and constitute the basis for further research on the possible neuroprotective role of BBR and other isoquinoline alkaloids. Hijazi et al. [42] emphasized that amyloid-beta-induced hyperexcitability of hippocampal inhibitory PV interneurons contribute to neuronal network dysfunction and thus memory impairment in a mouse model of AD. It seems that restoring the activity of these neurons can save spatial memory disorders. In our research, the administration of berberine had an effect on the improvement of memory in mice, which seems to correlate with immunohistochemical studies in which we conclude that the increase in the PV-IR of interneurons may have a significant impact on memory processes.

## 4. Materials and Methods

### 4.1. Reagents and Plant Material

The reagent grade solvents used for the preparation of extracts and the purification of BBR by CPC chromatography (methanol, methyl-*tert*-butyl ether, trietylamine and hydrochloric acid) were purchased from the Avantor Performance Materials (Gliwice, Poland). The HPLC analyses were performed using an HPLC grade acetonitrile, acetic acid, and millipore water from Merck (Darmstadt, Germany). The solvents used for the HPLC-MS-based quantitative analysis of BBR in mouse brains and plasma—water, acetonitrile, and formic acid—were obtained from J.T. Baker (Phillipsburg, NJ, USA). The standard of berberine (95% purity) used for the quantitative analysis was produced by Sigma Aldrich (St. Louis, MO, USA).

The plant material used in the study was collected in Nalaikh, Tuv aimag province, Mongolia, in July 2014. The geographical coordinates were as follows: V722+34 Nalaikh (47.8502412, 107.2502551). The overground parts were finely cut, dried in the air and shadow, and ground to a fine powder. The overground parts of *Berberis sibirica* were authenticated by prof. E. Ganbold from the Institute of Botany, Mongolian Academy of Sciences, and by Dr. Khurelbat Daariimaa, from the Mongolian National University of Medical Sciences in Ulan-Bataar, who keeps a voucher specimen (No. GD 0108) in the Laboratory of Pharmacognosy, Mongolian National University of Medical Sciences. The identification was performed on site based on the experience and the key to the vascular plants of Mongolia.

The sample of plant material is also kept by the authors in the Department of Pharmacognosy at the Medical University of Lublin, Poland.

### 4.2. Extraction and Chromatographic Isolation of Berberine from Plant Matrix by Centrifugal Partition Chromatography (CPC)

The powder obtained from the overground parts of *Berberis sibirica* was extracted by the ASE 100 extractor (Accelerated Solvent Extractor, Dionex, Sunnyvale, CA, USA) in the following conditions: solvent—methanol, temperature of extraction—90 °C, purge time—50 s, extraction time—5 min, number of cycles—5, flush volume—50%. Portions of ca. 20 g of the herb were extracted in 33 mL stainless steel vessels. The pressure was maintained at ca. 100 bar. The obtained extracts were joined and evaporated to dryness under reduced pressure on a rotary evaporator at 50 °C.

The isolation of BBR from the obtained extract was performed on a centrifugal partition chromatograph SCPC-250-L (Armen Instruments, Saint Ave, France) equipped in a quaternary pump, a 250 mL column, a UV detector, and a fraction collector. The isolation was performed in the pH-zone refining mode of operation, with the lower phase used as a stationary phase in the previously published conditions [18]. Briefly, the biphasic solvent system was composed of methyl-*tert*-butyl ether and water (1:1 *v*/*v*). The lower aqueous phase contained 10 mMol/L of hydrochloric acid, whereas the upper mobile phase contained 10 mMol/L of trietylamine. Ca 100 mg of the extract was dissolved in 5 mL of a 70:30 (*v*/*v*) mixture of lower to upper phases, but with no addition of trietylamine to the upper phase. The column was filled with an acidified lower phase at the rotation speed of 500 rpm and the flow rate of 20 mL/min. The mobile phase was pumped on the column together with the sample injection at the rotation speed of 1050 rpm and the flow rate of 5 mL/min. The separation was performed for 220 min, but the BBR used for the study was obtained within the first 30 min. The purity of the isolate was determined in the HPLC-UV analysis using the Shimadzu chromatograph (Kyoto, Japan) equipped in a quaternary pump (LC-20 CE), a degasser (DGU-20A), a column thermostat (CTO-10 AS VP), a PDA detector (SPD-M 20A), and the Kinetex RP-18 (250 mm × 4.6 mm, 5 µm) chromatographic column (Phenomenex, Torrance, CA, USA) in the following detection wavelengths: 210, 254, 280, 320, and 366 nm. The following gradient of acetonitrile with 2% of acetic acid (A) in 2% acetic acid was used: 0 min, 1% A; 40 min, 20% A; 60 min, 40% A; 70 min, 1% A. The run time was set at 70 min, the flow rate at 1 mL/min, and the thermostat temperature at 25 °C.

### 4.3. Animals

The experiments were carried out on naive male Swiss mice (Farm of Laboratory Animals, Warszawa, Poland) weighing 20–30 g. The animals were maintained under standard laboratory conditions (12-h light/dark cycle, room temperature 21 ± 1 °C) with free access to tap water and laboratory chow (Agropol, Motycz, Poland) in their home cages, and adapted to the laboratory conditions for one week. Each experimental group consisted of 7–9 animals. All behavioral experiments were performed between 8:00 a.m.and 3:00 p.m., and were conducted according to the National Institute of Health Guidelines for the Care and Use of Laboratory Animals and to the European Community Council Directive for the Care and Use of laboratory animals of 22 September 2010 (2010/63/EU), and approved by the local ethics committee.

### 4.4. Drugs

The berberine that was isolated from the methanolic extract of *Berberis sibirica* herb was transferred to a mortar and dissolved in saline with the addition of 0.2% DMSO. The compound was administered intraperitoneally (i.p.) at the concentration of 5 mg/kg of body weight. Fresh drug solutions were prepared on each day of experimentation. Control groups received injections of saline with 0.2% DMSO at the same volume and by the same route of administration.

### 4.5. Experimental Procedures

Memory-related responses were measured by the passive avoidance (PA) task. The apparatus of PA consisted of two-compartment acrylic box with a lighted compartment (10 × 13 × 15 cm) and darkened compartment (25 × 20 × 15 cm). The light chamber was illuminated by a fluorescent light (8 W) and was connected to the dark chamber, which was equipped with an electric grid floor. The entrance of animals to the dark box was punished by an electric foot shock (0.2 mA for 2 s).

The doses of BBR were selected based on our previous results [20], which show that this alkaloid exhibited memory enhancing properties in mice after an acute administration. Moreover, based on the results of locomotor activity tests, the dose that did not affect the locomotor activity of animals was selected for chronic treatment, to be sure that no disturbance occurs in the memory tests. BBR (2.5 or 5 mg/kg) or saline for the control group were administered intraperitoneally once a day for 7 days 15 min before the injection of saline. On the 8th day, BBR (2.5 or 5 mg/kg) or saline was administered i.p. 15 min before saline. 15 min after last injection the pre-test was conducted. During the pre-test, mice were placed individually into the light compartment and allowed to explore the light box. After 30 s, the guillotine door was raised to allow the mice to enter the dark compartment. When the mice entered the dark compartment, the guillotine door was closed and an electric foot-shock (0.2 mA) of 2 s duration was delivered immediately to the animal via the grid floor. The latency time for entering the dark compartment was recorded (TL1). If the mouse failed to enter the dark box within 300 s, it was placed into this dark box, the door was closed, and an electric foot-shock was delivered to the animal. In this case, the TL1 value was recorded as 300 s.

In the subsequent trial (retention), 24 h later, the same mice were again placed individually in the light compartment of the PA apparatus. After a 30 s adaptation period in the light (safe) chamber, the door between the compartments was raised and the time taken to re-enter the dark compartment was recorded (TL2). No foot-shock was applied in this trial. If the animal did not enter the dark compartment within 300 s, the test was stopped and TL2 was recorded as 300 s.

### 4.6. Immunohistochemistry and Antibodies

The brains from 30 male mice were stored in 10% buffered formalin (pH = 7) for 12 h at 4 °C, dehydrated in ethyl alcohol and embedded in paraffin blocks according to a previously described method [39]. Briefly, the paraffin blocks were cut into 5 µm-thick sections which were placed on silanized glass-slides (SuperFrostPlus, Braunschweig, Germany). To block the endogenous peroxidase activity, the sections were chilled and washed in 3% hydrogen peroxidase (20 min). The slides were then flushed twice with PBS (pH = 7.4) (15 min each time) and incubated in 2.5% normal goat serum (ImPRESS^TM^; MP-7451, Vector Labs, Burlingame, CA, USA) at room temperature (RT) for 20 min. The sections were incubated for 24 h at 4 °C with primary monoclonal rabbit antibodies raised against PV (1:2000; SWANT, PV25, Fribourg, Switzerland). The next day, the slides were washed in a washing buffer (2 × 15 min) and covered with anti-rabbit Ig (ImPRESS^TM^; MP-7451 Vector Labs, Burlingame, CA, USA) for 1 h. For the visualization of primary antisera, 3,3′-diaminobenzidine (ImmPACT^TM^DAB, SK-4105, Vector Labs, Burlingame, CA, USA) chromogen was used. A working solution of DAB was applied onto the slides, and the process was monitored under a light microscope. Finally, the slides were rinsed with distilled water. Moreover, counterstaining (for 20 min) with Mayer’s hematoxylin was performed. After washing in distilled water, the slides were dehydrated in an ethyl alcohol series, cleared in xylene, mounted in DPX (Sigma-Aldrich, St. Louis, MO, USA) and cover slipped. The slides were viewed under a light microscope (Axiolab, Zeiss, Jena, Germany) connected to a digital camera. From each animal, approx. 25–30 of immune-stained sections for PV were analyzed. No less than one hundred of PV-IR neurons in CA1, CA2, CA3, and DG were viewed and counted. The specificity of the antibodies used was verified using a negative control in which primary antibodies were replaced with the same concentrations of appropriate non-immune IgG.

### 4.7. The HPLC-ESI-QTOF-MS Quantitative Analysis of Mice Brains

50 µL of each plasma sample (*n* = 3) were vortexed with 50 µL with cold acetonitrile for 60 s. Then, 50 µL of water was added and mixed again and the obtained solutions were filtered through nylon syringe filters (0.2 µm pore diameter) produced by Merck (Darmstadt, Germany). The mouse brains (*n* = 3) were homogenized on ice with 100 µL of acetonitrile using a PRO 200 Bio-Gen Series tissue homogenizer (Pro Scientific Inc., Oxford, UK). After the homogenization, 100 µL of water was added, and the samples were centrifuged for 30 min at 3000 rpm on a centrifuge (Eppendorff Centrifuge 5427R, Hamburg, Germany). Then, the supernatant was collected, filtered through a nylon syringe filter (pore diameter of 0.2 µm) and subjected to a HPLC-MS analysis. The concentration of BBR was also determined for the isolated hippocampus (*n* = 3). They were subjected to a similar procedure as the brains, but the volume of acetonitrile used for the homogenization was 50 µL, and the one used for water was also 50 µL. The volumes of the added solvents were included in the calculations of the BBR concentration in the biological material.

The HPLC-ESI-QTOF-MS/MS instrument used in the study was produced by Agilent Technologies (Santa Clara, CA, USA) and was composed of a 1200 Series chromatograph (that contained a binary pump, a degasser, an autosampler, a column thermostat and a PDA detector) and a 6500 Series ESI-QTOF-MS/MS mass spectrometer. A gradient of acetonitrile with the addition of 0.1% formic acid (A) in a 0.1% aqueous solution of formic acid was used as follows: 0 min, 10% A; 10 min, 40% A; 12 min, 40% A; 17 min, 95% A; 20 min, 95% A; 21 min, 10% A. The run time was set at 35 min, the flow rate at 0.2 mL/min, and the temperature of thermostat at 25 °C. The following conditions for the detection of BBR in the biological samples were introduced on a mass spectrometer, the gas and sheath gas temperatures: 350 and 325 °C, respectively, the gas and sheath gas flows: 12 L/min, the capillary voltage: 3500 V, the nebulizer pressure: 30 psig, the skimmer voltage: 65 V, the fragmentor voltage: 110 V, the collision energies: 20 and 40 V. The experiments were recorded in the positive ionization mode in the *m*/*z* range of 100–1000 u by the dedicated software: Mass Hunter Workstation (version B.08.00).

### 4.8. Statistical Analysis

The statistical analysis was performed using a one-way analysis of variance (ANOVA). A post-hoc comparison of means was carried out with the Tukey’s test for multiple comparisons, when appropriate. The data were considered statistically significant at a confidence limit of *p* < 0.05. ANOVA analysis with Tukey’s post-doc test were performed using GraphPad Prism version 5.00 for Windows, GraphPad Software, San Diego, CA, USA, www.graphpad.com (accessed on 10 November 2020).

For the memory-related behaviors, the changes in PA performance were expressed as the difference between the retention and training latencies and were taken as a latency index (LI). LI was calculated for each animal as the following ratio: LI = TL2 − TL1/TL1.

TL1—the time taken to enter the dark compartment during the training; TL2—the time taken to re-enter the dark compartment during the retention.

The statistical analysis of immunohistochemical studies was performed using a one-way analysis of variance (ANOVA) followed by the Tukey’s test for variables that meet the conditions of normal distribution (*p* < 0.05). A Kruskal–Wallis test was used for comparisons of nonparametric results (*p* < 0.05).

## 5. Conclusions

In this study berberine was isolated from *Berberis sibirica* using the centrifugal partition chromatography technique operating with a biphasic solvent system composed of methyl-*tert*-butyl ether and water (1:1 *v*/*v*), with the addition of 10 mMol/L of trietylamine and hydrochloric acid, each. The alkaloid at the purity of 96.5% was used for behavioral, immunohistochemical, and quantitative studies on a HPLC-ESI-QTOF-MS/MS spectrometer. The results showed an increased concentration of berberine in the hippocampus calculated as 38.12 ± 2.12 ng per organ after the administration of 5 mg/kg b.w. to mice. The chronic injection of berberine has showed significant memory-improving activities in the passive avoidance test.

Based on the obtained results of the preliminary study, berberine can be treated as a drug candidate supporting the therapy of neurodegenerative diseases, especially AD. In conclusion, an increase of PV-IR neurons after the administration of BBR may suggest the effect of this alkaloid on neuronal Ca^2+^ metabolism and constitutes the basis for further research on the possible neuroprotective role of BBR and other isoquinoline alkaloids.

## Figures and Tables

**Table 1:** Average numbers of PV-IR neurons in the CA1, CA2, and CA3 hippocampal fields and DG of mice from the control and experimental groups: 5 and 2.5 mg/kg BBR (i.p.).

	CA1 (Neurons PV-IR)	CA2 (Neurons PV-IR)	CA3 (Neurons PV-IR)	DG (Neurons PV-IR)
Control group	2.9 ± 1.09 ^A^	3.1 ± 1.2 ^A,B,^*	4.3 ± 1.1 ^A^	2.5 ± 0.8
2.5 mg/kg group	3.2 ± 1.1 ^A^	3.5 ± 1.1 ^B^	4.5 ± 0.8 ^A^	2.7 ± 0.8
5 mg/kg group	10.4 ± 2.2 ^B^	10.1 ± 1.7 ^B^	12.4 ± 2.05 ^B^	2.6 ± 0.7

Different letters represent statistically significant differences; ANOVA, *p* < 0.05. * average compared with the Kruskal–Wallis test, *p* < 0.05.
